# Spatial Relationship Quantification between Environmental, Socioeconomic and Health Data at Different Geographic Levels

**DOI:** 10.3390/ijerph110403765

**Published:** 2014-04-03

**Authors:** Mahdi-Salim Saib, Julien Caudeville, Florence Carre, Olivier Ganry, Alain Trugeon, Andre Cicolella

**Affiliations:** 1 French National Institute for Industrial Environment and Risks, Parc Technologique Alata, BP 2, 60550 Verneuil-en-Halatte, France; E-Mails: Julien.CAUDEVILLE@ineris.fr (J.C.); Florence.CARRE@ineris.fr (F.C.); Andre.CICOLELLA@ineris.fr (A.C.); 2 University of Picardie Jules Verne; 33 rue St Leu, Amiens 80039, France; 3 University Hospital of Amiens; Place Victor Pauchet Amiens 80054, France; E-Mail: Ganry.olivier@chu-amiens.fr; 4 Regional Observatory of Health and Social Issues in Picardie (OR2S); 3, rue des Louvels, Amiens 80036, France; E-Mail: Alain.trugeon@or2s.fr

**Keywords:** health inequalities, socioeconomic status, exposure indicator, geographic level, MAUP, Geographically Weighted Regression

## Abstract

Spatial health inequalities have often been analyzed in terms of socioeconomic and environmental factors. The present study aimed to evaluate spatial relationships between spatial data collected at different spatial scales. The approach was illustrated using health outcomes (mortality attributable to cancer) initially aggregated to the county level, district socioeconomic covariates, and exposure data modeled on a regular grid. Geographically weighted regression (GWR) was used to quantify spatial relationships. The strongest associations were found when low deprivation was associated with lower lip, oral cavity and pharynx cancer mortality and when low environmental pollution was associated with low pleural cancer mortality. However, applying this approach to other areas or to other causes of death or with other indicators requires continuous exploratory analysis to assess the role of the modifiable areal unit problem (MAUP) and downscaling the health data on the study of the relationship, which will allow decision-makers to develop interventions where they are most needed.

## 1. Introduction

Analyzing the relationship between the environment and health has become a major issue for public health in France as forecasted by the national plans for health and environment (NPHE). Two priority areas were selected during the first NPHE: (1) preventing health risks related to the quality of resources and to chemicals and (2) developing environmental health through research, expertise, training and information. In 2009, the second NPHE was prepared from the perspective of the upcoming conference on health and the environment organized by the World Health Organization. Two main axes were prioritized: (1) identifying and managing geographic areas where hotspot exposures to substances present in air, soil, water, and foods resulting from anthropic activities suspected of generating potentially increasing risks to human health and (2) reducing environmental health inequalities. Thus, environmental health inequality has become a substantial topic that guides policy developments in France. To address this aim, there is an urgent need for tools that can quantify the spatial relationships between the environment, socioeconomics and health and that can highlight areas with strong inequalities. 

Health inequalities are a quite recent study topic. Previous studies were essentially based, at an individual level, on specific surveys [1,2] and, at a spatially aggregated level (administrative unit), on specific regions [3,4]. At a regional scale, data are often available at a fine level or resolution. This allows for building environmental, socioeconomic and health indicators at different spatial scales; for example, Salmond *et al.* [5] built a new census-based index of deprivation based on the smallest possible geographical areas. 

Regarding health data, there are strict privacy rules for individual-level health data that prohibit their public release. Aggregated data are only available at the geographic level, from which disclosure and reconstruction of patient identity are impossible. In France these census units could be regions or counties. This aggregation unfortunately results in incidence or mortality rates that can be unreliable over small and/or sparsely populated areas. This effect, known as the “small number problem” [6], should be corrected for an accurate evaluation of health-environment relationships. 

Several authors have already addressed the spatial relationships between health data and environmental data. One of the issues faced by spatial epidemiologists and for exposure assessment is the combination of data measured for very different spatial scales and with different levels of reliability. In reality, the analysis of cancer mortality maps is often hindered by the presence of noise caused by unreliable extreme rates computed from sparsely populated geographic units. A number of approaches have been developed to improve the reliability of risk estimates [7,8]. The most commonly used are Bayesian methods [9], which are commonly referred to as the BYM model. Bayesian methods prohibit any change of scales, an operation that is easily conducted within the framework of kriging. Goovaerts and Gebreab [10] conducted a simulation-based evaluation of the performance of geostatistical and full Bayesian disease-mapping models, and they found that the geostatistical approach yielded smaller prediction errors and more precise and accurate probability intervals and that it allowed for better discrimination between counties with high and low mortality risks. 

Poisson kriging, in this context, presents a spatial methodology that allows for filtering the noise caused by the small number problem and enables the estimation of mortality risk and the associated uncertainty at different spatial scales. This approach has been implemented to modeling cancer risk by a number of authors: Oliver *et al.* [11] studied cases of cancer in children under fifteen years of age, and Goovaerts and collaborators considered lung cancer [12,13], breast cancer [14,15], prostate cancer [16], cervical cancer [17], and pancreatic cancer [18], and all found it to be relevant for this particular problem.

Selection of scale is perhaps the most important factor in creating and analyzing a relationship between environmental exposure and health outcomes [19]. This issue is similar to the modifiable area unit problem (MAUP), a term introduced by Openshaw [20,21]. The MAUP can cause differences in the analytical results of the same input data compiled under different zoning systems [22,23].

The present study aims to evaluate spatial relationships at three levels of aggregation: the IRIS level, an intermediate scale (the grid level), and the county level between health outcomes (mortality attributable to cancer) initially aggregated to the county level, district socioeconomic covariates, and exposure data modeled on a regular grid. The approach is illustrated using age-adjusted lip, oral cavity and pharynx, and pleural cancer mortality rates over the period 2000–2009 for the Picardy region. The deprivation index and trace metal exposure indicators are used as putative risk factors.

## 2. Materials and Methods

### 2.1. Study Area

**Figure 1:**
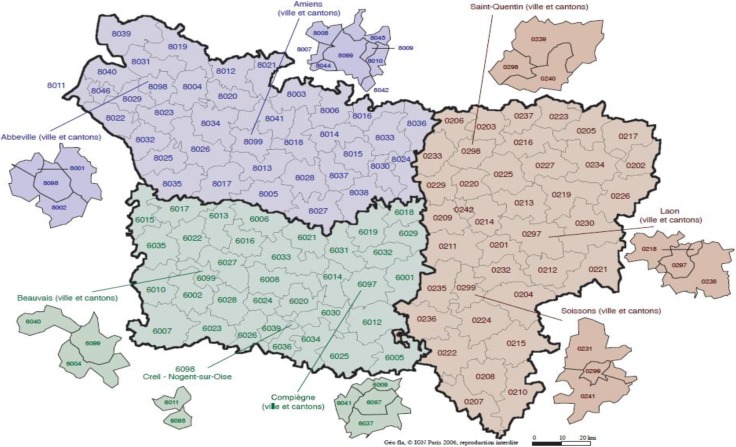
Map of the study area.

The region of Picardy covers an area of roughly 19,500 km^2^ and is located between the North Artois, the Ile-de-France in the south, the Bay of the Somme to the west and the East Champagne. It covers the departments of Somme, Oise and Aisne. The urbanization rate in this region is far below the national average (60.4% *versus* 74% for the entire country). The agricultural sector provides more than 4% of French agricultural production. It also has significant industrial activity through which fine chemicals and specialty chemicals account for nearly 15% of jobs and the vehicle industry comprises 40% of industrial employment (26.5% of assets employed in industry *versus* 19.5% at the national level). Three administrative or statistical spatial units, of different sizes, were considered: IRIS (the smallest administrative units in Picardy, 2,129 units) with irregular sizes and shapes, 64 km^2^ grid cells (308 units) that are all squares of same size, and counties (112 units) with irregular sizes and irregular shapes. Figure 1 shows the counties of the study area.

### 2.2. Data

#### 2.2.1. Exposure Indicators

The environmental indicators (inhalation and ingestion) used were those described in Caudeville *et al.* for building GIS-based modeling platforms for quantifying human exposure to chemical substances [24]. The exposure indicators integrate soil, water, air, food, demographic and behavioral geo-referenced data to construct population exposure doses and associated risks at a fine resolution (1 km^2^ grid).

**Table:** 

0201 Anizy-le-Château	6001 Attichy	8001 Abbeville Nord
0202 Aubenton	6002 Auneuil	8002 Abbeville Sud
0203 Bohain-en-Vermandois	6004 Beauvais Sud-Ouest	8003 Acheux-en-Amiénois
0204 Braine	6005 Betz	8004 Ailly-le-Haut-Clocher
0205 La Capelle	6006 Breteuil	8005 Ailly-ser-Noye
0206 Le Catelet	6007 Chaumont-en-Vexin	8006 Albert
0207 Charly	6008 Clermont	8007 Amiens Ouest
0208 Château Thierry	6009 Compiègne Nord	8008 Amiens Nord-Ouest
0209 Chauny	6010 Le Coudray-Saint-Germer	8009 Amiens-Nord-Est
0210 Condé-en-Brie	6011 Creil-Nogent-sur-Oise	8010 Amiens-Est
0211Coucy-le-Château Auffrique	6012 Crépy-en-Valois	8011 Ault
0212 Craonne	6013 Crèvecoeur-le-Grand	8012 Bernaville
0213 Crécy-sr-Serre	6014 Estrées-Saint-Denis	8013 Boves
0214 La Fère	6015 Formerie	8014 Bray-sur-Somme
0215 Fère-en-Tardenois	6016 Froissy	8015 Chaulnes
0216 Guise	6017 Grandvilliers	8016 Combles
0217 Hirson	6018 Guiscard	8017 Conty
0218 Laon Nord	6019 Lassigny	8018 Corbie
0219 Marle	6020 Liancourt	8019 Crécy-en-Ponthieu
0220 Moy-de-l’Aisne	6021 Maignelay-Montigny	8020 Domart-en-Ponthieu
0221 Neufchatel-sur-Aisne	6022 Marseille-en-Beauvaisis	8021 Doullens
0222 Neuilly-Saint-Front	6023 Méru	8022 Gamaches
0223 Le Nouvion-en-Thiérache	6024 Mouy	8023 Hallencourt
0224 Oulchy-le-Château	6025 Nanteuil-le-Haudoin	8024 Ham
0225 Ribemont	2026 Neuilly-en-Thelle	8025 Homoy-le-Bourg
0226 Rozoy-sur-Serre	2027 Nivillers	8026 Molliens-Dreuil
0227 Sains-Richaumont	6028 Noailles	8027 Montdidier
0229 Saint-Simon	6029 Noyon	8028 Moreuil
0230 Sissonne	6030 Pont-Sainte-Maxence	8029 Moyenneville
0231 Soissons-Nord	6031 Ressons-sur-Matz	8030 Nesle
0232 Vailly-sur-Aisne	6032 Ribécourt-Dreslincourt	8031 Nouvion
0233 Vermand	6033 Saint-Just-en-Chaussée	8032 Oisemont
0234 Vervins	6034 Senlis	8033 Péronne
0235 Vic-sur-Aisne	6035 Songeons	8034 Picquigny
0236 Villers-Cotterets	6036 Chantilly	8035 Poix-de-Picardie
0237 Wassigny	6037 Compiègne-Sud-Est	8036 Roisel
0238 Laon-Sud	6039 Montataire	8037 Rosières-en-Santerre
0239 Saint-Quentin-Nord	6040 Beauvais-Nord-Ouest	8038 Roye
0240 Saint-Quentin-Sud	6041 Compiègne Sud-Oest	8039 Rue
0241 Soissons-Sud	6097 Compiègne	8040 Saint-Valery-sur-Somme
0242 Tergnier	6098 Creil	8041 Villers-Bocage
0297 Laon	6099 Beauvais	8042 Amiens Sud-Est
0298 Saint-Quentin		8043 Amiens Sud-Ouest
0299 Soisson		8044 Amiens Nord
		8046 Friville-Escarbotin
		8098 Abbeville
		8099 Amiens
Laon(ville et cantons) comprend les cantons 0218,0238 et 0297	Compiègne(ville et cantons) comprend les cantons 6009, 6037, 6041 et 6097	Amiens(ville et cantons) comprend les cantons 8007,8008, 8009, 8010 8042,8044,8045 et 8099
Saint-Quentin(ville et cantons) comprend les cantons 0239,0240 et 0298	Creil-Nogent-sur-Oise comprend les cantons 6011 et 6098	Abbeville(ville et cantons) comprend les cantons 8001,8002, et 8098
Soissons(ville et cantons) comprend les cantons 0231,0241 et 0299	Beauvais(ville et cantons) comprend les cantons 6004, 6040 et 6099	

Trace elements (nickel-Ni, cadmium-Cd, and lead-Pb) were modeled within the Picardy region [25] for various exposure pathways: atmospheric contaminant inhalation and ingestion of soil, vegetation, meat, eggs, milk, fish and drinking water.

#### 2.2.2. Deprivation Index (SE)

The deprivation index used was developed by Rey [26] and was built at the French census block (IRIS) using the following socioeconomic data: the median household income, the percentage of high school graduates in the population aged 15 years and older, the percentage of blue-collar workers in the active population, and the unemployment rate. The deprivation index was also constructed for the county. For each county, the deprivation index was calculated as the population-weighted average score for all of the IRISes in the county.

#### 2.2.3. Health Data

The health data came from the Regional Health Observatory of Picardy [27], where the age-adjusted mortality rates are calculated for each county from 2000 to 2009. Table 1 shows the cumulative, maximum and minimum number of mortality and age-adjusted rates per 100,000 person-years by county from 2000 to 2009.

**Table 1:** Cumulative, maximum and minimum number of mortality and age-adjusted rates per 100,000 person-years by county, 2000–2009.

Cancer Mortality	Numbers of Cases	Age-adjusted Rates Per 100,000 Person-years
**Lip, oral cavity and pharynx cancer mortality**		
Cumulative	1,327	16.26
Minimum	1	2.81
Maximum	128	37.4
**Pleural cancer mortality**		
Cumulative	263	3.78
Minimum	0	0
Maximum	18	11.94

**Figure 2:**
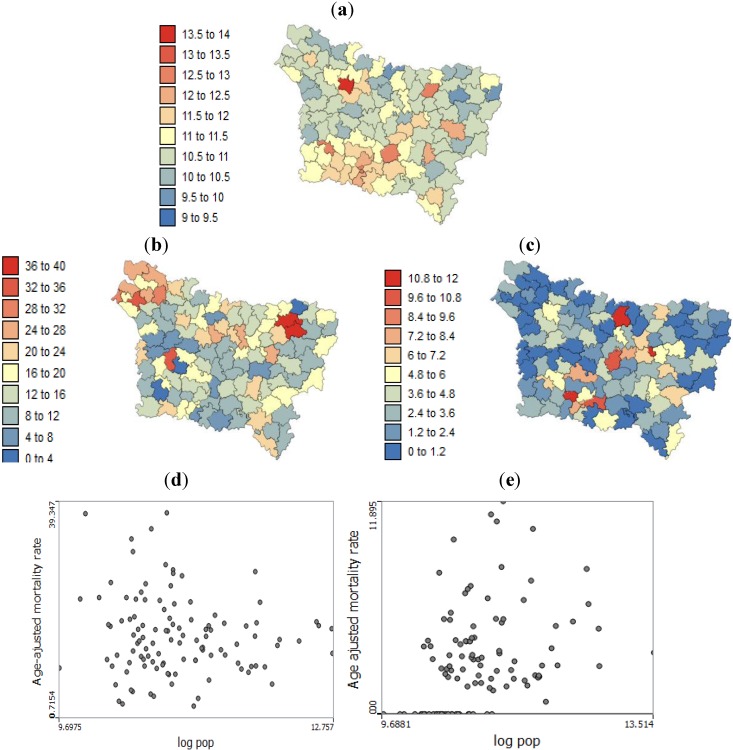
(**a**) Map of log population density. Geographic distribution of age-adjusted mortality rates per 100,000 person-years recorded over the period 2000–2009 for: (**b**) lip, oral cavity and pharynx; (**c**) pleura cancer mortality. The bottom scatter plots illustrate: (**d**) the age-adjusted mortality rates for lip, oral cavity and pharynx cancers plotted against population density and (**e**) the age-adjusted mortality rates of pleura cancers plotted against population density.

Figure 2 shows the spatial distribution age-adjusted lip, oral cavity, pharynx, and pleura cancer mortality per 100,000 person-years. It should be noted that (1) the population is not evenly distributed throughout the study area and (2) the age-adjusted rate calculated from the less-populated counties tend to be less reliable. This implies that the interpretation of the map must be carried out with caution. The scatter plot at the bottom of Figure 1 illustrates this effect, well-known as the “small number problem”.

Table 2 presents the different spatial scales of measurement and the approaches used to homogenize spatial coverage.

**Table 2:** Spatially resolved data types and approaches used to homogenize spatial coverage.

Indicator	Variables	Sources	Spatial Scale or Resolution	Spatial Operation
Socioeconomic	SE: Deprivation index	French census Rey *et al.* [26]	Vector data from the IRIS.	Spatial population-weighted aggregation
Exposure	F1: Exposure inhalation indicator F2: Exposure ingestion indicator	Caudeville *et al*. [24,25]	Raster data of 1 km^2^ grid	Spatial aggregation
Health	Lip, oral cavity and pharynx cancer mortality	Regional Health Observatory of Picardy [27]	Vector data from the county database	Poisson kriging
	Pleural cancer mortality			

### 2.3. Methods

#### 2.3.1. The Geostatistical Approach: Correcting Small Numbers and Estimating the Corresponding Risk at Different Spatial Scales

To correct for the instability attributable to the small number problem, a number of algorithms have been developed that aim at estimating risk. The geostatistical approach, in this context, presents an interesting alternative; it conducts the noise filtering and allows for risk estimation along with the associated uncertainty at different scales. This section provides a brief overview of the geostatistical methodology for estimating risk values. See Goovaerts [17] for more details about this approach.

The cancer mortality count *d*(*ν_α_*) within a county *ν_α_* is interpreted as the realization of a random variable *D*(*ν_α_*) that is Poisson distributed with a parameter (expected number of counts) that is the product of the population size *n*(*ν_α_*) by the local risk *R*(*ν_α_*). *R*(*ν_α_*) might be thought of as a noise-filtered mortality rate for area *ν_α_*, which we also refer to as the mortality risk. It is estimated by using a variant of kriging with nonsystematic errors known as Poisson kriging [28].

The mortality risk and the associated kriging variance for an area *ν_α_* are estimated as:

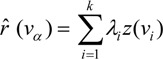



Kriging variance is computed as follows:

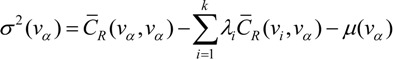

where *x* represents either an area *ν_α_* (ATA kriging). The kriging weights (*λ_i_*) and the Lagrange parameter μ (*ν_α_*) are computed by solving the Poisson kriging system of equations:

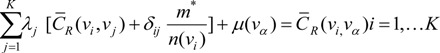


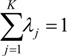

where *δ_ij_* = 1 if i = j and 0 otherwise. The “error variance” term, m^*^/n(*ν_i_*) leads to smaller weights for rates measured over smaller population sizes. The covariance *C_R_* (*ν_i_*, *ν_j_*) is approximated as the population-weighted average of the point-support covariance *C_R_* (*h*) computed between any two discrete locations between the areas *ν_i_* and *ν_j_*.

#### 2.3.2. Spatial Autocorrelation

Global Moran’s I was calculated for all of the explanatory variables as well as for the dependent variables within three spatial structures to determine the role of spatial representation using global spatial autocorrelation. The Global Moran’s I spatial autocorrelation statistic measures the self-similarity of a spatial variable’s value as a function of adjacency [29], using a first-order Queen’s case spatial weight matrix and 999 permutations.

#### 2.3.3. Exploring the Relationships between Health, Environment and Socioeconomic Factors

Analyses of correlations between health data and putative factors are traditionally performed using a global or “aspatial” regression model, under the implicit assumption that the impact of variables is constant over the entire study area. This assumption is likely unrealistic for large areas, which can display large geographic variations. Fotheringham and colleagues developed Geographically Weighted Regression (GWR) to explore spatial non-stationarity and map statistics to visualize the spatial patterns of the relationships between dependent and independent variables [30,31,32]. 

##### Aspatial Regression

The explanatory power of SE and exposure indicators was first investigated using the following multiple linear regression model:

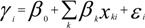

where *γ* is the kriging risks estimate for observation *i*, *β*_0_ is the intercept, *β_k_* is the regression coefficient (slope) of each factor *x_k_* and *ε_i_* is the error term. To account for the reliability of the kriged risks in the regression, each observation receives a weight that is the reciprocal of the kriging variance [33].

##### Geographically Weighted Regression.

In geographically weighted regression, the regression is conducted within local windows centered around each observation. The regression coefficients are thus location-dependent:

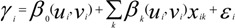



Within each window, observations are weighted according to their proximities to the center of the window. A variety of distance decay functions are available. In this paper, we used the XX function, which is characterized by a bandwidth that corresponds to the distance beyond which the weight rapidly approaches zero.

The bandwidth is estimated by minimizing the AICc value:

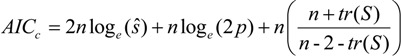



where *n* is the number of observations in the dataset, 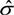 is the estimate of the standard deviation of the residuals, and *tr*(*S*) is the trace of the hat matrix. For more information on the theory and practical application of GWR, the reader is referred to Fotheringham *et al*. [34].

## 3. Results

### 3.1. Poisson Kriging for Health Indicator

**Figure 3:**
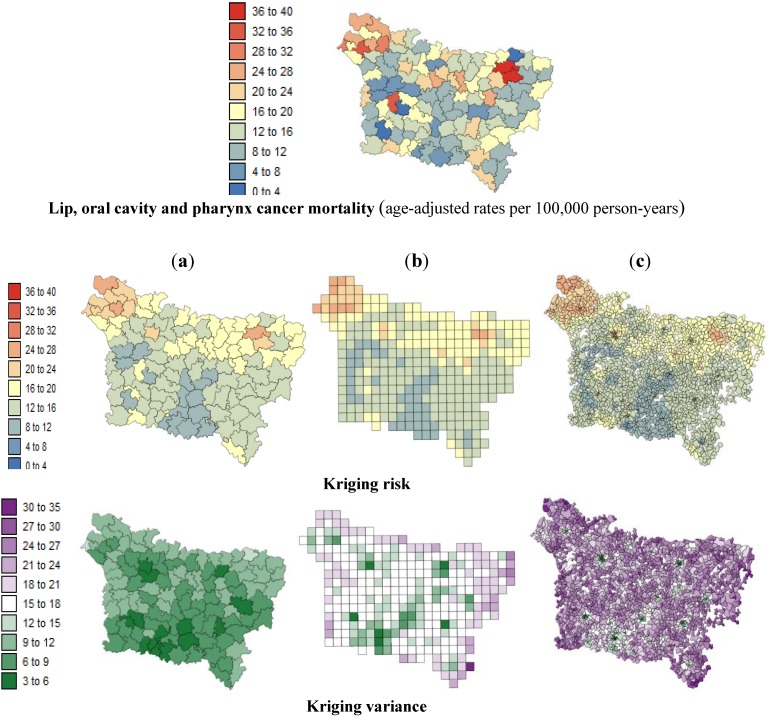
Maps of the lip, oral cavity and pharynx cancer mortality risk estimates and the corresponding prediction variance computed by Poisson kriging at three spatial scales: (**a**) county level; (**b**) grid level and (**c**) IRIS level.

**Figure 4:**
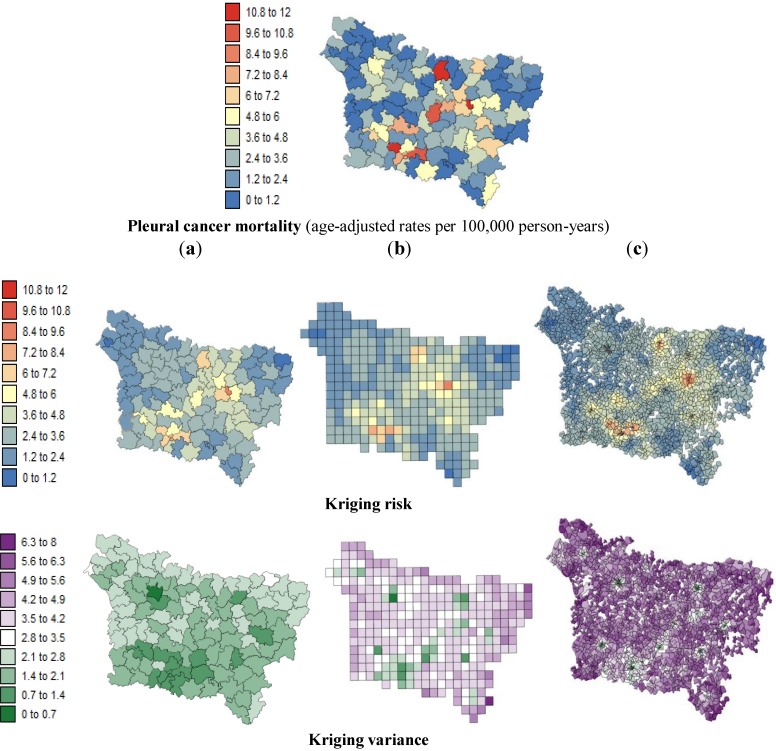
Maps of the pleural cancer mortality risk estimates and the corresponding prediction variances computed by Poisson kriging at three spatial scales: (**a**) county level; (**b**) grid level; and (**c**) IRIS level.

Figure 3 and Figure 4 show the risk values with the corresponding prediction variance estimated by Poisson kriging at: (a) the county level; (b) the grid level and (c) the IRIS level. All maps are substantially smoother than the original rate map because the noise caused by small population sizes has been filtered. These maps allow a better visualization of areas of higher risks: the lip, oral cavity and pharynx cancer mortality rates vary between 2.81 and 37.40 per 100,000 person-years. After the application of Poisson kriging, the minimum rate increased from 2.81 to 8.87 deaths/100,000 person-years, and the maximum rate of 37.40 decreased to 25.14 deaths per 100,000 person-years. We can note, for instance, that the high rates recorded in sparsely populated counties such as Sains-Richaumont (37.40 deaths/100,000 person-years), north of the Aisne department, are strongly smoothed (24.46 deaths/100,000 person-years). The highest rate recorded in a densely populated county (*i.e.*, Abbeville North county-26.60 deaths/100,000 person-years) remained nearly the same after smoothing (24.90 deaths/100,000 person-years). Zero pleural cancer mortality rates recorded in sparsely populated counties were also smoothed, leading to minimum values of 1.00 deaths/100,000 person-years.

The maps of the kriging variance indicate the higher reliability of risks estimated in densely populated areas such as Amiens, Beauvais, Saint Quentin, and Abbeville. The variance of the risk estimates decreased as the geographic unit area increased: from the IRIS level to the grid level and then to the county level (Table 3).

The risk estimates are characterized by positive spatial autocorrelation within the three spatial scales (*p* ≤ 0.05) but display low levels of statistically significant spatial autocorrelation at the IRIS level in comparison with the grid and county levels (Table 3). In this case, the counties are internally homogeneous in terms of mortality according to the risks estimated by kriging.

**Table 3:** Summary statistics for health indicators after applying Poisson kriging.

Lip. Oral Cavity and Pharynx Cancer Mortality				
Estimation Type	Mean	Min	Max	Morans’I
	**County Level**			
Kriging risk	15.59	8.88	25.14	0.65 (0.001)
Kriging variance	8.36	1.87	13.42	
	**Grid Level**			
Kriging risk	15.32	8.31	25.92	0.78 (0.001)
Kriging variance	16.06	2.81	30.09	
	**IRIS Level**			
Kriging risk	15.35	7.38	26.56	0.96 (0.001)
Kriging variance	22.52	4.1	33.24	
**Pleural Cancer Mortality**				
**Variables**	**Mean**	**Min**	**Max**	**Moran’s I**
	**County Level**			
Kriging risk	3.16	1	8.72	0.52 (0.001)
Kriging variance	1.92	0.43	3.07	
	**Grid Level**			
Kriging risk	2.99	0.87	8.48	0.62 (0.001)
Kriging variance	3.65	0.63	6.67	
	**IRIS Level**			
Kriging risk	3.21	0.87	9.04	0.93 (0.001)
Kriging variance	4.99	0.89	7.32	

### 3.2. Spatial Aggregation for the Explanatory Variables

The mean values of the explanatory variables under each of the three spatial structures are similar (Table 4). The variables F1 (exposure inhalation indicator) and F2 (exposure ingestion indicator) display greatly reduced variability under different spatial structures. The variable SE has the highest levels of variability. In contrast, the SE index is affected by the use of different spatial structures. The lowest average was −4.5 at the county level in comparison with −7.32 at the IRIS level, and the variance decreased with increasing aggregation, unlike with F1 and F2, for which the lowest and strongest averages were somewhat similar for the three spatial scales.

**Figure 5:**
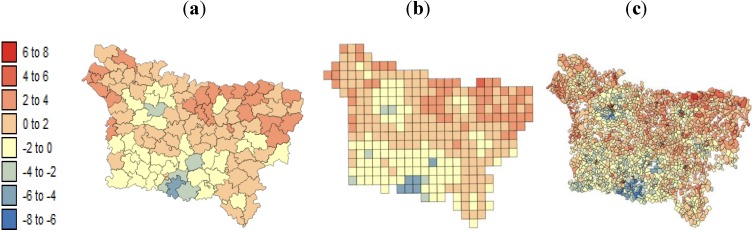
Maps of the deprivation index computed at three spatial scales: (**a**) county level; (**b**) grid level; (**c**) IRIS level.

**Figure 6:**
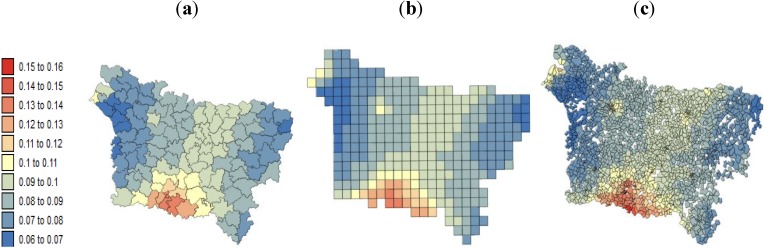
Maps of the *exposure inhalation indicator* (F1) aggregated at: (**a**) county level; (**b**) grid level; (**c**) IRIS level.

All of the variables were characterized by positive spatial autocorrelation within the three spatial scales at levels of *p* ≤ 0.05. For F1 and F2, the Moran’s I values of the three spatial structures were similar (Table 4); however, the F1 and F2 were not affected by the use of different spatial structures. This is explained by the fact that the exposure indicator presented a homogeneous distribution within each county (the original resolution of exposure indicator data was a grid (15 × 10 km); see Caudville *et al.* [24,25]. Figure 5 and Figure 6 show maps of the SE index and the F1 aggregated at (a) county level, (b) grid level and (c) IRIS level.

**Table 4:** Summary statistics for the explanatory variables.

Variables		Mean	Min	Max	Variance	Moran's I
		**County Level**				
SE: Deprivation index		0.61	−4.5	3.48	2.84	0.63(0.001)
F1: Exposure inhalation indicator		0.08	0.06	0.13	0.0002	0.81(0.001)
F2: Exposure ingestion indicator		0.27	0.27	0.39	0.002	0.61(0.001)
		**Grid Level**				
SE: Deprivation index		0.58	−5.1	4.1	3.02	0.70(0.001)
F1: Exposure inhalation indicator		0.08	0.06	0.13	0.0002	0.88(0.001)
F2: Exposure ingestion indicator		0.27	0.28	0.48	0.002	0.61(0.001)
		**IRIS Level**				
SE: Deprivation index		0.48	−7.3	−8	4.62	0.55(0.001)
F1: Exposure inhalation indicator		0.08	0.06	0.15	2,00E−04	0.91(0.001)
F2: Exposure ingestion indicator		0.26	0.31	0.68	0.003	0.65(0.001)

### 3.3. Aspatial Regression

Table 5 shows the correlation coefficient between each covariate and mortality rates before and after noise-filtering using Poisson kriging. Filtering the noise because of the small number problem clearly enhanced the explanatory power of the covariates: the proportion of variance explained (adjusted R^2^) increased by nearly one order of magnitude: lip, oral cavity and pharynx cancer mortality, 0.11 to 0.26, and pleural cancer mortality, 0.11 to 0.25. The uncertainty attached to the risk estimates can be accounted for by weighting each estimate according to the inverse of its kriging variance, leading to correlation coefficients and adjusted R^2^ values that were slightly larger for pleural cancer mortality and a slightly lower mortality rate for lip, oral cavity and pharynx cancers.

Application of the linear model for lip, oral cavity and pharynx cancer mortality data explained a moderate proportion of the total variance (adjusted R^2^ = 0.22 and 0.19) at the county level and grid level, respectively. This proportion was lower when the analysis was conducted at the IRIS level (adjusted R^2^ = 0.11). It is noteworthy that the correlation coefficients for the SE factor were always significant for the different aggregation levels and were higher at the county level than at the IRIS level, which was the expected result because aggregation is known to increase the strength of correlation [20,35]. Linear association between SE index and cancer mortality has been demonstrated in other works [36,37,38,39].

**Table 5:** Results of the correlation analysis.

Lip. Oral Cavity and Pharynx Cancer Mortality				
**Regression Model**	SE	F1	F2	Adjusted R^2^
	**County-level correlation**			
Age-adjusted rate	0.32 *	−0.11	0.04	0.11
Kriging risk	0.53 *	−0.27	0.06	0.26
Kriging risk (weighted)	0.49 *	−0.26	0.03	0.22
	**Grid-level correlation (64 km)**			
Kriging risk	0.49 *	−0.28	0.03	0.24
Kriging risk (weighted)	0.43 *	−0.26	0.01	0.19
	**IRIS-level correlation**			
Kriging risk	0.37 *	−0.21	0.01	0.15
Kriging risk (weighted)	0.32 *	−0.13 *	−0.03	0.11
**Pleural Cancer Mortality**				
**Regression Model**	SE	F1	F2	Adjusted R^2^
	**County-level correlation**			
Age-adjusted rate	−0.13	0.35 *	0.03	0.11
Kriging risk	−0.18	0.51 *	0.02	0.25
Kriging risk (weighted)	−0.16	0.52 *	−0.01	0.28
	**Grid-level correlation (64 km)**			
Kriging risk	−0.18	0.47 *	0.04	0.20
Kriging risk (weighted)	−0.17	0.49 *	0.03	0.24
	**IRIS-level correlation**			
Kriging risk	−0.01	0.46 *	0.06	0.22
Kriging risk (weighted)	0.04	0.50 *	0.05	0.28

Notes: * Significant at α = 0.01; SE: Deprivation index; F1: Exposure inhalation indicator; F2: Exposure ingestion indicator.

The model explains a moderate proportion of the total variance when the dependent variable *y* is pleural cancer mortality for different levels of aggregation. The adjusted R^2^ ranges between 0.24 and 0.28, with significant correlation coefficients of up to 0.5 for F1. The results showed the consistency of the association between trace metal inhalation exposure and pleural mortality across the different aggregation levels. This is explained by the fact that the pleural mortality presented a homogeneous distribution within each county and the exposure indicator was not affected by the use of different spatial structures. Pleural mesothelioma is one of four types of mesothelioma, but it accounts for approximately 75 percent of all diagnoses of asbestos-related cancers. The disease starts in the pleura, the soft protective tissue surrounding the lungs, which can be attributed directly to its cause: repeated or heavy occupational exposure to airborne asbestos fibers. However, Peterson suggests that a significant number of cases of this cancer are apparently not asbestos-related and that even in the absence of asbestos, other agents can induce malignant mesothelioma [40]. Some types of nanoparticles, especially those containing nickel, could also be a risk of pleural diseases [41].

### 3.4. Geographically Weighted Regression (GWR)

In the aspatial analysis, we implicitly assumed that the impact of covariates was constant across the study area. This assumption is likely unrealistic for large areas, which can display substantial geographic variation in socioeconomic and environmental conditions. Therefore, the global statistics reported by this traditional regression model could potentially hide a number of interesting local relationships. The question is then to examine whether there are any meaningful spatial variations in these relationships.

**Figure 7:**
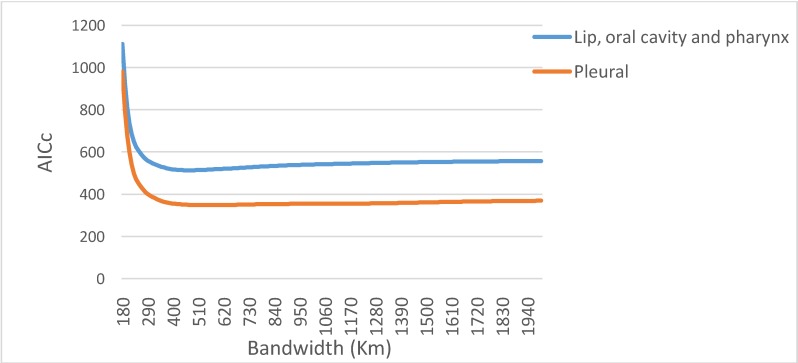
Impact of bandwidth size on the AICc of geographically weighted regression for each cancer.

County-level data were used to optimize the bandwidth of the GWR distance decay function. Figure 7 shows the relationship between AICc and bandwidth size for the two types of cancer. The following bandwidths were found to be optimal: lip, oral cavity and pharynx cancer mortality (47 km), and pleural cancer mortality (54 km). The local regression was based on the following number of closest observations, which represented 15%–20% of the data: 22 for the county level, 39 for the grid level, and 426 for the IRIS level. A comparison of the AICc values suggests that all of the GWR models outperformed the global model (Table 6). 

These results strongly suggest that the relationships between cancer (lip, oral cavity and pharynx-pleural) mortality and the environmental and deprivation indexes are not stationary but instead vary over the study area. The application of GWR clearly enhances the explanatory power of the covariates for the three spatial levels: the proportion of variance explained (adjusted R^2^) is almost doubled (Table 6).

**Figure 8:**
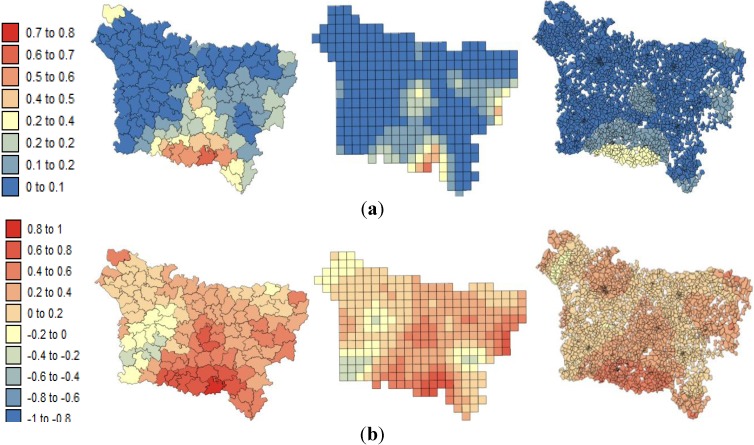
Results of the geographically weighted regression applied to the lip mortality kriged rates: (**a**) maps of the proportions of variance explained by deprivation index (R^2^); (**b**) maps of the local correlation coefficients with the deprivation index.

**Figure 9:**
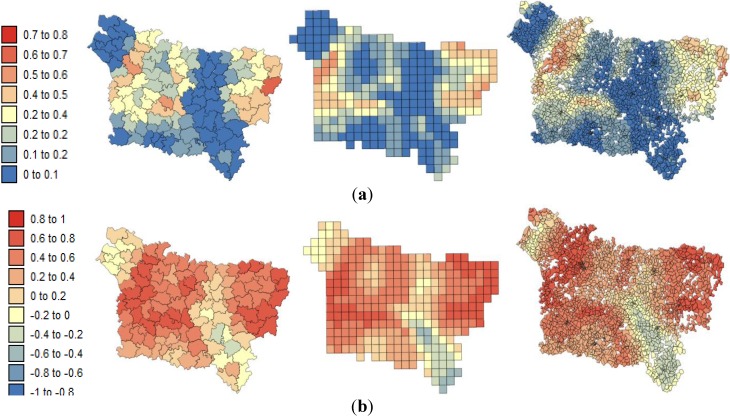
Results of the geographically weighted regression applied to the pleural cancer mortality kriged rates: (**a**) maps of the proportion of variance explained by the exposure inhalation indicator (R^2^); (**b**) maps of the local correlation coefficients with the exposure inhalation indicator.

Figure 8 and Figure 9 show the results of the geographically weighted regression applied to the county, grid and IRIS data. Two statistics are displayed for each spatial scale: (a) the proportion of variance explained (R^2^) and (b) the local correlation coefficient.

The three spatial scales share the same southeast-northwest trend in the explanatory power of the local regression models for lip, oral cavity and pharynx cancer mortality: the lower mortality values in the south are better explained by the SE index than is the higher risk recorded in the northwest. As a recall, the largest R^2^ observed in the south (Figure 8 a) corresponds to areas with low SE index variation. Similar to the global model (Table 5), GWR led to local correlation coefficients and R^2^ values that were higher at the county level than at the IRIS level.

The lower pleural cancer mortality values are better explained in areas of low F1 variation (Figure 9a). The maps of the local correlation coefficients and the R^2^ values also present the same spatial structure over the different spatial coverages of aggregation: lower in the west оf tһе Aisne department and higher in the north of the Oise department. Very similar results were obtained for the different spatial scales.

**Table 6:** Comparison of local and global regression models at the three spatial scales.

Lip. Oral Cavity and Pharynx Cancer Mortality			
Regression model	Bandwidth Size	Adjusted R2	AICc
	**County-level correlation**		
Global model	47 km	0.22	567.00
Local model		0.52	513.47
	**Grid-level correlation (64 km)**		
Global model	47 km	0.19	1,530.76
Local model		0.48	1,280.26
	**IRIS-level correlation**		
Global model	47 km	0.11	10,932.00
Local model		0.21	10,112.32
**Pleural Cancer Mortality**			
**Regression Model**	Bandwidth Size	Adjusted R2	AICc
	**County-level correlation**		
Global model		0.28	374.35
Local model	54 km	0.48	348.09
	**Grid-level correlation (64 km)**		
Global model		0.24	931.65
Local model	54 km	0.49	803.08
	**IRIS-level correlation**		
Global model		0.28	6,219.21
Local model	54 km	0.46	5,852.26

## 4. Discussion

Our results substantiate the work on noise filtering described in the introduction section from Oliver *et al.* [11] and Goovaerts *et al.* [12,18]. Indeed, we found the following: (1) the mean risk values estimated under each of the three spatial structures were similar; (2) the IRIS risk estimates were non-negative; (3) and their sums were equal to the original county risk. Although the disaggregation of cancer data on a small scale is somewhat arbitrary, in particular when it does not take into account secondary information to guide this downscaling, the approach should facilitate the analysis of the relationships between health data and the putative covariates (*i.e.*, environmental, socioeconomic, behavioral or demographic factors) that are typically estimated for different spatial scales. These covariates can potentially be subsequently used as secondary information in the kriging, leading to more detailed risk maps at finer scales [42].

The other issue was the so-called modifiable areal unit problem (MAUP), for which different geographic scales can lead to inconsistent results for relationships analysis. For example, the mortality rate reported at (1) the county level requires an aggregated deprivation index at the same resolution, and this aggregation obscures the intra-county variation and thus the relationship and (2) the IRIS level, at which the disaggregation leads to a large variance in estimated risk. Exploratory methods, such as the univariate Moran’s can serve as indications of the potential effect of the MAUP in the study how relationships based on the homogeneity and heterogeneity of spatial data are affected by the study level and may affect the ability of the study to detect a relationship [43].

In this study, very similar results were obtained for the different spatial scales between:

- pleural cancer mortality and the exposure indicator F1.
- lip, oral cavity, pharynx cancer mortality and the SE index.

Whereas other studies on the relationship between heath and deprivation showed that the use of spatial representations other than the census tract produced different analytical results [35,44], the significant association between lip, oral cavity, pharynx cancer mortality and the SE index estimated using the county structure were stronger than they were under the IRIS structure. 

This difference between the significant correlation coefficients is the result of aggregation because the aggregation level is known to increase the strength of correlation [20]. Future work could provide tools to exhaust all possible aggregations and generate empirical frequency distributions of the statistical estimates that could be used to evaluate the sensitivity of results to aggregation effects.

Based on the results obtained, we can confirm that the presence of this significant statistical association was likely not induced by the use of a particular geography. At the three spatial scales, the strongest correlation coefficients were found where low deprivation was associated with low lip, oral cavity and pharynx cancer mortality and where low environmental pollution was associated with low pleural cancer mortality. 

## 5. Conclusions

This paper presents an approach for evaluating spatial relationships between health outcomes (mortality attributable to cancer) initially aggregated at the county level, district socioeconomic covariates, and exposure data modeled on a regular grid. The approach was illustrated using age-adjusted lip, oral cavity and pharynx, and pleural cancer mortality rates measured over the period 2000–2009 for the Picardy region. The deprivation index and trace metal exposure indicators were used as putative risk factors. For the different spatial scales, the strongest associations were found where low deprivation was associated with low lip, oral cavity and pharynx cancer mortality and where low environmental pollution was associated with low pleural cancer mortality. However, applying this approach to other areas, for other causes of death, or with other indicators always requires exploratory analysis to assess the role of the MAUP and downscaling health data in the study of the relationships that will allow decision-makers to develop interventions where they are the most needed. 
